# Label-Free Potentiometry for Detecting DNA Hybridization Using Peptide Nucleic Acid and DNA Probes

**DOI:** 10.3390/s130202267

**Published:** 2013-02-07

**Authors:** Tatsuro Goda, Ankit Balram Singi, Yasuhiro Maeda, Akira Matsumoto, Masaki Torimura, Hiroshi Aoki, Yuji Miyahara

**Affiliations:** 1 Institute of Biomaterials and Bioengineering, Tokyo Medical and Dental University, 2-3-10 Kanda-Surugadai, Chiyoda, Tokyo 101-0062, Japan; E-Mails: goda.bsr@tmd.ac.jp (T.G.); ankitsingi007@gmail.com (A.B.S.); yas.maeda.bsr@tmd.ac.jp (Y.M.); matsumoto.bsr@tmd.ac.jp (A.M.); 2 National Institute of Advanced Industrial Science and Technology (AIST), 16-1 Onogawa, Tsukuba, Ibaraki 305-8569, Japan; E-Mail: torimura-masaki@aist.go.jp

**Keywords:** PNA, DNA, hybridization, electrostatic energy, label-free biosensor, potentiometry

## Abstract

Peptide nucleic acid (PNA) has outstanding affinity over DNA for complementary nucleic acid sequences by forming a PNA-DNA heterodimer upon hybridization via Watson-Crick base-pairing. To verify whether PNA probes on an electrode surface enhance sensitivity for potentiometric DNA detection or not, we conducted a comparative study on the hybridization of PNA and DNA probes on the surface of a 10-channel gold electrodes microarray. Changes in the charge density as a result of hybridization at the solution/electrode interface on the self-assembled monolayer (SAM)-formed microelectrodes were directly transformed into potentiometric signals using a high input impedance electrometer. The charge readout allows label-free, reagent-less, and multi-parallel detection of target oligonucleotides without any optical assistance. The differences in the probe lengths between 15- to 22-mer dramatically influenced on the sensitivity of the PNA and DNA sensors. Molecular type of the capturing probe did not affect the degree of potential shift. Theoretical model for charged rod-like duplex using the Gouy-Chapman equation indicates the dominant effect of electrostatic attractive forces between anionic DNA and underlying electrode at the electrolyte/electrode interface in the potentiometry.

## 1. Introduction

The development of biosensors with high sensitivity and selectivity is of paramount importance for clinical diagnostics, genomics, and drug discovery. Techniques that could distinguish analytes without the need for labels, *i.e.*, label-free sensing, are of great interest because they would not only significantly reduce the cost and time needed for sample preparation, but would also eliminate issues related to modification of target molecules. As such, numerous studies have been undertaken to develop label-free sensors that directly detect the binding of a target. Surface plasmon resonance (SPR) has long been considered the golden standard for label-free biological detection, and has achieved sensitivities below nanomolar range for direct, non-amplified DNA detection [1,2]. Electrochemical techniques have also been developed for nucleic acids biosensing with high sensitivity and selectivity to DNA target [3]. The signal generation is mainly based on the modulation of electron transfer activity of labeled or free redox indicators upon hybridization or strand displacement by target recognition. Another promising platform for unlabeled sensing is nanomaterials-based field-effect transistors (FETs) [4–8]. The binding of the specific ligand or complementary DNA modifies the electric field surrounding the device, enabling direct electronic detection as low as femtomolar in a salt free solution and picomolar in solutions at physiological ionic strength. However, nanowire/nanotube devices suffer from alignment and reproducibility issues.

Another approach to gain the sensitivity is the selection of ligands as a capturing element. Typical bioreceptors for electrical biosensing are nucleic acids, proteins, amino acids, antibodies and enzymes. This analyte-bioreceptors interaction yields an electrical signal at the interface. Peptide nucleic acid (PNA), originally developed as a gene-targeting drug, has demonstrated remarkable hybridization properties towards complementary oligonucleotides by increasing thermal stability of the hybrids [9–14]. Neutral PNA is advantageous for facilitating duplex formation with a complementary DNA sequence. The use of short PNA oligomers as capture probes can result in hybridization with DNA at lower salt concentrations [15,16]. While, DNA-DNA hybridization suffers from dominant charge repulsion in a low salt solution because of the abundant negative charges in the phosphate backbone. For hybridization to occur, a minimum of 10 mM electrolyte is required to screen out the electrostatic repulsion. These advantages have aided to develop PNA-based sensors for DNA detection using various techniques in recent years [17]. Uno *et al.* have previously demonstrated increased electrical signals on direct DNA detection using PNA-immobilized ion-sensitive FET (ISFET) [18]. The electrical readout of negative charges from captured DNA at the interface using FET devices allows direct detection of complementary DNA without the need for reporter molecules and labeling [19–21].

The aim of the present work is to investigate the sensitivity for DNA target using PNA or DNA probes immobilized on the SAM surface in potentiometry. We focused on using a high input impedance electrometer for label-free detection of the DNA hybridization by the direct electrical readout of captured DNA charges [22]. Likewise extended-gate FET biosensors, hybridization of nucleic acids is transduced to a change in the electrical characteristic at the solution/electrode interface using the electrometer [23]. We obtained the potential shift at the interface of a 10-channel gold microelectrodes array in the hybridization buffer and during rinse. We expected that the detection of PNA-DNA hybridization is more effective than the DNA-DNA hybridization because of the increased affinity by forming a stable PNA-DNA heterodimer.

## 2. Experimental Section

The synthetic PNA and DNA oligomers were purchased from Fasmac (Atsugi, Kanagawa, Japan) and Tsukuba Oligo Services (Tsukuba, Ibaraki, Japan), respectively. The sequences used are listed in Table 1. The PNA and DNA probes were modified by 5′-Cys-O-(CH_2_)_2_-O- and 5′-SH-(CH_2_)_6_-, respectively, for making covalent attachment onto the gold electrodes. PNA was dissolved in the aqueous solution of 0.1 wt% trifluoroacetic acid prior to use. All the other reagents with extra-high grades were purchased and used without further purifications.

To make a reaction chamber on the chip, a 2.5-mm radius glass ring was immobilized on the microelectrode array chip with a thermosetting epoxy resin for 2 h at 120 °C. The chamber including the gold electrodes was cleaned by dipping in 1 M NaOH aq for 5 min and subsequently in 1 M HCl aq for 5 min. The clean gold electrodes were treated with 10 μM PNA or DNA probe in 1× TE buffer (10 mM Tris-HCl, 1 mM EDTA-2Na) for 12 h. Thereafter, the electrodes were treated with 100 μM 6-mercapto-1-hexanol (MCH) for another 12 h.

A 10-channel gold microelectrode array (Towa Tech., Iwata, Shizuoka, Japan) with an immobilized glass ring was connected to the high input impedance electrometer (6517B, Keithley, Cleveland, OH, USA). The electrical potential was set at 0 V versus an Ag/AgCl reference electrode (in 3.3 M KCl aq with a salt bridge) throughout the measurement. Following stabilization of electrical potential in 0.1× Dulbecco's Phosphate Buffered Saline (DPBS) for more than 2 h, Hybridization with DNA target was allowed in 5× Saline-Sodium Citrate (SSC) buffer (750 mM NaCl, 75 mM sodium citrate) for 20 min at 37 °C, thereafter the electrodes were rinsed in 0.1× DPBS for 20 min at 37 °C. DNA target concentration was changed from 10^−16^ M to 10^−10^ M in a stepwise manner in logarithmic scale. For negative control random and non-complimentary DNA were used. In this study, *p* < 0.01 calculated by a two-tailed paired Student's T-test were treated as statistically significant.

## 3. Results and Discussion

**Figure 1.**
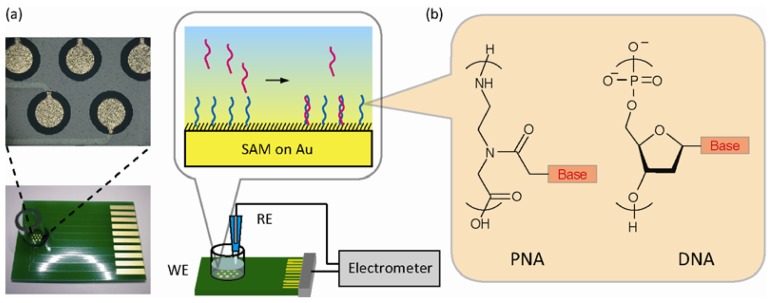
Schematic representation of label-free electrical detection of DNA hybridization using PNA or DNA probes. (**a**) Electrometer-based potentiometric setup for monitoring changes in charge density upon capturing DNA target at the solution/microelectrodes interface. The images indicate the gold microelectrodes array. (**b**) Differences in the backbone structure and electrostatic nature of PNA and DNA.

Figure 1(a) shows the experimental setup of the label-free, reagentless, and real-time potentiometry to sense DNA target hybridization with the PNA or DNA probes immobilized on the microelectrode array. The specific duplex formation based on Watson-Crick base-pairing alters charge density (*ΔQ*) owing to the intrinsic negative charge of captured DNA within the electrical double layer served as a capacitor (*C*_DL_) at the solution/electrode interface. In some situations, double layer capacitance is also changed by the binding event (*ΔC*_DL_). This is mainly due to displacement of water molecules and ions at the interface upon target binding. Biomolecular binding decreases relative permittivity of the double layer (2–5 for biomolecules versus 80 for water), both decreasing capacitance. Therefore, the overall potential shift upon hybridization can be represented as:

(1) $$\Delta V=\left(Q+\Delta Q\right)/\left(C_{\text{DL}}+\Delta C_{\text{DL}})-(Q/C_{\text{DL}}\right)$$

Namely, following the formation of PNA-DNA or DNA-DNA duplexes at the interface, some regions of the cylinder-like double-helix conformation may overlap with the electrical double layer. The DNA molecules located outside the electrical double layer fail to generate an interfacial potential as a result of the charge-screening effect by mobile counterions supplied by the external electrolytes in the buffer solution. The electrolyte concentration determines the solution Debye length (κ^−1^) that represents characteristic charge-screening effect of the solution as follows:

(2) $$\kappa^{-1}={\left({ɛ}_{\text{r}}{ɛ}_{0}{\text{k}}_{\text{B}}\text{T}\;/\;2{\text{z}}^{2}{\text{q}}^{2}\text{I}\right)}^{1/2}$$

where ε_r_ is the relative permittivity of the electrolyte solution, ε_0_ is the vacuum permittivity, k_B_ T is the Boltzmann energy, z is the valency of the ions in the electrolyte, q is the elementary charge, and I is the ionic strength of the electrolyte. Theoretical calculation of the Debye length was 0.35 nm and 2.6 nm for 5× SSC buffer and 0.1× DPBS, respectively, at 37 °C. PNA molecules are the neutral analogue of DNA [Figure 1(b)]. DNA has a deoxyribose backbone, whereas PNA is composed of repeating *N*-(2-aminoethyl)glycine units linked by amide bonds. The various purine and pyrimidine bases are linked to the backbone by methylenecarbonyl bonds. PNAs are depicted like peptides, with the *N*-terminus at the first (5′) position and the *C*-terminus at the 3′. Since the backbone of PNA contains no charged phosphate groups, the binding affinity between PNA and DNA strands is stronger than that of DNA-DNA duplex due to the lack of electrostatic repulsion. On the other hand, the electrical neutrality makes long PNA oligomers rather hydrophobic, impairing the solubility in aqueous media [24].

It is important to control the surface density of the immobilized probes for hybridization detection because the exclusion volume effect between the adjacent probes prevents efficient hybridization [25]. The surface density of the PNA and DNA probes was determined by cyclic voltammetry [26] and chronocoulometry techniques [27], respectively (Table 2). The densities of the PNA probes indicate the distance between the adjacent probes of 2.1 to 2.8 nm. These values are approximately consistent with the diameter of double-stranded DNA of 2.4 nm. The surface densities of the DNA probes were a bit lower than those of the PNA probes with the calculated lateral probe distance of about 3.6–3.8 nm. The results suggest that the DNA probes repel each other through steric and electrostatic restrictions. The neutral PNA may aid to increase the probe density because of the absence of electrostatic repulsion between the immobilized probes. The lateral probe distance should be larger than 2.4 nm for efficient hybridization. Whilst, for sensitive detection of hybridization, the probe density should be the highest in the value range where high hybridization efficiency is maintained. The rest of the gold electrode was covered with MCH SAM to remove nonspecifically adsorbed probes on the electrode and to minimize nonspecific adsorption of DNA molecules during the hybridization process.

**Figure 2.**
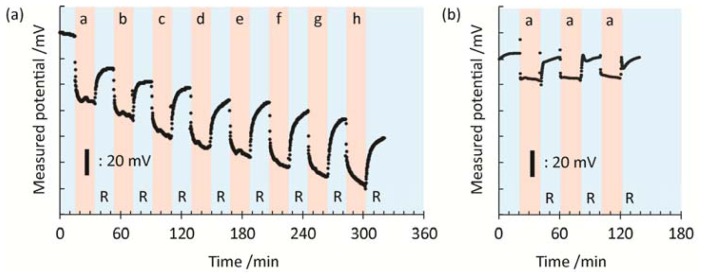
(**a**) Time-course of potential changes at the solution/electrode interface by applying DNA target with increasing concentration in 5× SSC buffer and by rinsing with 0.1× DPBS in stepwise manner to the gold electrode modified with MCH SAM containing the 20-mer PNA probes. (**b**) Stability of measured potential with repeated cycles of blank-rinse solutions. DNA target concentration: **a**. blank; **b**. 100 aM; **c**. 1 fM; **d**. 10 fM; **e**. 100 fM; **f**. 1 pM; **g**. 10 pM; **h**. 100 pM. R: rinsing.

Figure 2 represents the time course of the potential response during the hybridization/rinse measurements in a stepwise manner using the microelectrode in the potentiometry. First, we recorded the potentials in the blank hybridization buffer solution (5× SSC) and in the rinse buffer (0.1× DPBS) as the standard voltages. Then, different concentrations of DNA target were introduced from the most diluted one to the most concentrated one in a stepwise manner. The average voltage response during the last 3 min before changing the solutions was compared at the different concentrations of DNA target. Changes in the potential in the solutions of DNA target (“b–h” in Figure 2) are essentially derived from an increase in the negative charge density at the interface because of the specific and nonspecific bindings of DNA target onto the sensor surface. Note that the charge density is also changed by the ionic strength and composition of the buffer solution. While, the recovery of the potential during rinsing (“R” in Figure 2) is mainly caused by the removal of weakly or nonspecifically bound DNA target from the electrode surface. The shapes of the signal in the target and rinse solutions represent association and dissociation processes of DNA target which can be theoretically modeled by the Langmuir equation. The gradual changes in the potential are mainly caused by the association and dissociation processes with slow time constant which can be interpreted as a weak nonspecific interaction of DNA target with the sensor surface. The specific hybridization reaction between the probe and target occurs within a shorter timeframe. Therefore, the incubation and rinsing periods of 20 min are enough for extracting the changes in the charge density generated by the hybridization.

**Figure 3.**
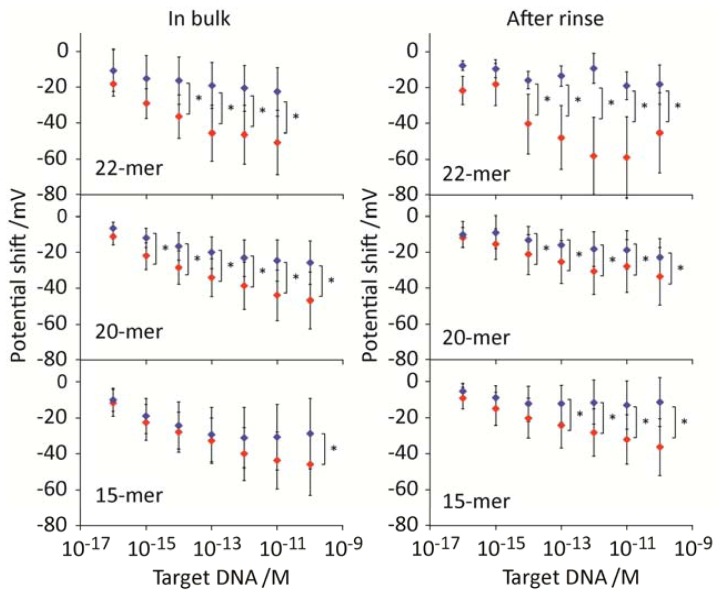
Potential shifts as a function of DNA target concentrations for the electrodes modified by PNA probes (22-mer, 20-mer, and 15-mer) and MCH SAM in bulk and after rinse conditions. Colors in red and blue represent the signals from DNA target and non-target, respectively. (* *p* < 0.01).

We obtained the potential variations as a function of DNA target concentration for the PNA probes both in the hybridization buffer containing DNA target and after rinse (without DNA target condition) (Figure 3). The PNA-DNA hybridization at 22-mer probe produced voltage shifts of 40–50 mV in the hybridization buffer, whereas the potential shifts were at 40–60 mV during rinse. The potential shifts were all to the negative direction, indicating the direct readout of negative innate charges of trapped DNA molecules at the interface. Non-complimentary DNA produced voltage shift at 20 mV both in hybridization buffer and during washout, respectively, indicating the detection of relatively high amount of nonspecific adsorption onto the electrode. Yet, the potential shifts following hybridization showed statistically significant differences at the DNA concentration of 10 fM both in the hybridization buffer and during rinse, owing to the comparison of data from multiple sensorgrams using the microelectrodes array system. Trends in the potential shift were similar in the systems for 22-mer, 20-mer, and 15-mer PNA probes, but the sensitivity for the hybridization detection decreased by decreasing the probe length. The loss of the sensitivity is strongly related to the reduced number of Watson-Crick base pairs between the PNA probes and DNA targets.

**Figure 4.**
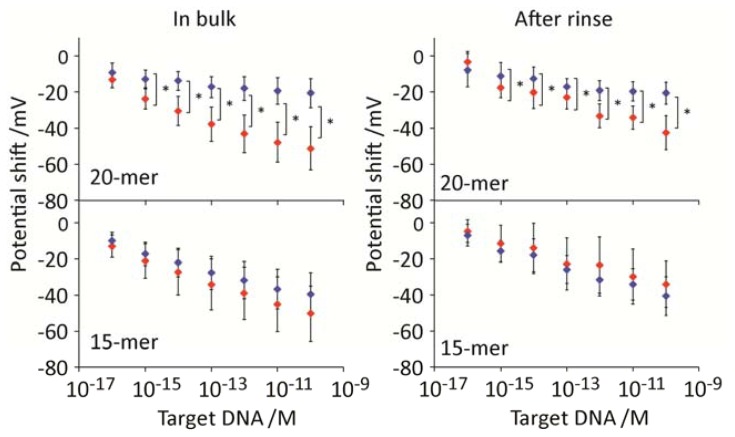
Potential shifts as a function of DNA target concentrations for the electrode modified by DNA probes (20-mer and 15-mer) and MCH SAM in bulk and after rinse conditions. Colors in red and blue represent the signals from DNA target and non-target, respectively. (* *p* < 0.01).

To compare the sensitivity between PNA and DNA probes, we conducted control experiments on hybridization using DNA probes with identical sequences to PNA. Figure 4 shows the potential shift as a result of DNA-DNA hybridization. Following introduction of DNA target solution to the electrodes array, negative potential shifts were observed in the concentration-dependent manner. For 20-mer DNA probe, statistically significant signals were obtained at the concentrations higher than 10^−15^ M both during the hybridization buffer condition and after rinse. While, no distinct signal changes were observed on the 15-mer DNA probes. This is the clear contrast on the increased affinity by using PNA probes for capturing the short DNA target (15-mer). In other words, PNA probe improved the affinity for short oligonucleotides compared with DNA one in potentiometry. However, for oligonucleotide with the length greater than 20-mer, there was no clear contrast on the sensitivity both in the high (hybridization buffer) and low (rinse buffer) ionic strength solutions.

PNA has better the affinity for base pairing over DNA so that PNA probes have contributed to increase the sensitivity for hybridization detection with complementary DNA in optical and electrochemical biosensing [28–31]. In contrast to our expectation, the signal enhancement was not so distinct in potentiometry especially for the detection of longer oligonucleotides. Additional unspecified observation was the significant potential shifts for target detection in the 5× SSC hybridization buffer. This extremely high ionic strength decreases the Debye length to 0.35 nm that corresponds to the distance of single base in the double helix. Under such condition of dominant charge screening, it may not be plausible to readout innate charges of DNA target in upright orientation after hybridization with the probe. One possible reason to answer the question is the electrostatic interaction between DNA and underlying electrode surface within the electrical double layer. Since the surface potential of MCH SAM formed on the gold electrodes is positive at no DC bias voltage versus Ag/AgCl reference electrode [32,33], DNA as an anionic polyelectrolyte is attracted onto the electrode. Indeed, Rant *et al.* have demonstrated electrical manipulation of orientation of immobilized DNA duplex on a charged gold surface [34–36]. The possibility for electrostatic adsorption of surface-immobilized dsDNA in various ionic strength solutions was evaluated by calculating electrostatic energy over thermal fluctuation using the charged rod-like dsDNA model. The rod is located at the distance of 0.7 nm, which corresponds to the thickness of MCH SAM above the electrode, and the length was determined by the number of base pairs and by the internucleotide spacing in dsDNA (0.34 nm). The Gouy-Chapman equation provides the diffusive potential distribution (Φ) under the dominant screening effect in a salt solution as follows:

(3) $$\Phi =\left(2{\text{k}}_{\text{B}}\text{T}\;/\;\text{q})\text{In}\;((1+\gamma \exp(-\kappa \text{d})\right)\;/\;\left(1-\gamma \exp(-\kappa \text{d}))\right)$$

with:

(4) $$\gamma =\tanh\left(\text{q}\Phi_{0}/4{\text{k}}_{\text{B}}\text{T}\right)$$

where d denotes the distance to the surface and Φ_0_ is the surface potential (50 mV). The electrostatic energy (E) of the DNA-rod depends on its tilt angle (θ) relative to the surface and is calculated by a summation over all point charges i as:

(5) $$\text{E}=\sum_{\text{i}}{\text{q}}_{\text{eff}}\;\Phi \left({\text{Z}}_{\text{i}}\right)$$

where q_eff_ is the effective charge of each DNA-DNA base pair (=−0.24 × 2q) [37].

**Figure 5.**
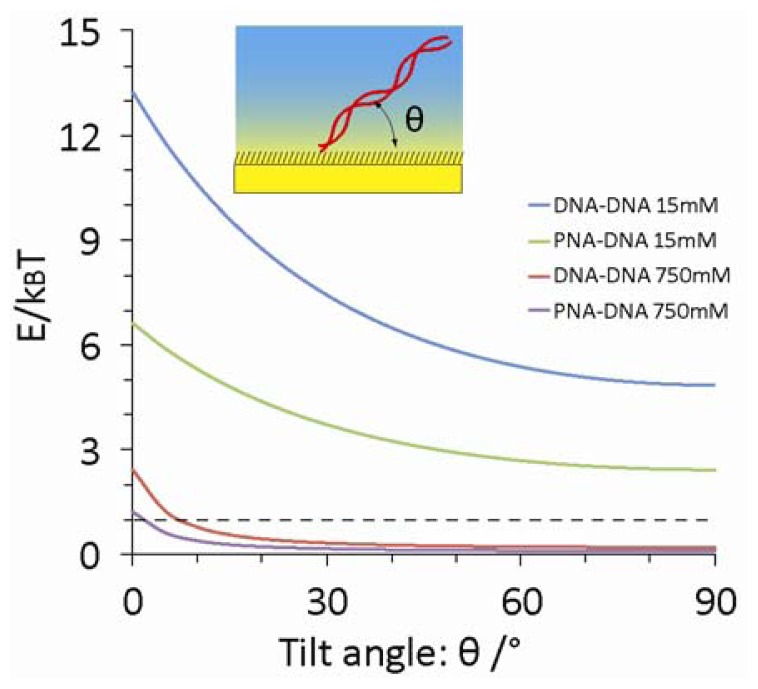
Electrostatic energy for 20-mer PNA-DNA or DNA-DNA duplexes using the charged rod-like model under the attractive Gouy-Chapman potential above the positive surface potential (Φ0 = 50 mV). Concentrations of monovalent salt in solution are 15 mM (rinse buffer: 0.1× DPBS) and 750 mM (hybridization buffer: 5× SSC).

Figure 5 displays the electrostatic energy in terms of k_B_T for surface-immobilized duplex in 5× SSC and in 0.1× DPBS using above equations. The calculation clearly shows that the electrostatic energy is higher than thermal fluctuation of the molecule (E/k_B_T > 1) even in 5× SSC buffer at the tilt angles below 8° and 3° for DNA-DNA and PNA-DNA duplexes, respectively. This model also indicates that the electrostatic interaction decreases with decreasing the number of charges and probe length. Therefore, the 15-mer PNA-DNA heterodimer has the weakest electrostatic interaction with the electrode surface. Schuhmann *et al.* have demonstrated the importance of controlled orientation of DNA probes on a SAM-coated electrode by surface potential for successful determination of DNA hybridization using electrochemical impedance spectroscopy [38]. In contrast, neutral PNA probes have no electrostatic interaction with the electrode that compromises the sensitivity for the hybridization detection through the potentiometry. In this study, this electrostatic attractive force explains successful electrical readout of DNA target even in the hybridization buffer. Also, the electrostatic interaction may accumulate free DNA molecules in the solution toward the sensor surface. This phenomenon locally increases the apparent target concentration at the interface, allowing highly sensitive detection in the low concentrations of DNA target. Relatively high noise levels observed in the potentiometry may be attributed to the nonspecific electrostatic accumulation of free DNA molecules onto the electrode. Due to the limitation to modulate the surface potential in electrostatic potentiometry, it is quite difficult to confirm this phenomenon. Assembling the mixed SAM with a dithiol as a ternary component may reduce background noises by improving surface coverage and defects of backfilling SAM [39,40]. Introducing anti-fouling thols instead of MCH for making SAM may be also effective to decrease noises by preventing nonspecific adsorption [41,42]. Another reason for influencing on the results is the dramatic conformational change of the single-stranded probes from globular shape to the rigid rod-like duplex with the persistence length of about 45–50 nm [43], which is a clear contrast to that of 1–2 nm for single stranded DNA [44]. Indeed, the flexibility change of PNA probe upon duplex formation with DNA target can be detected in electrochemical biosensing [26]. Because the potentiometric sensor detects changes in the charge density and capacitance at the interface as shown in Equation (1) so that the conformational changes should dramatically influence on the sensor signal. The chain flexibility of PNA and polyelectrolytic nature of DNA might change macromolecular conformation of PNA and DNA probes at the interface.

## 4. Conclusions

Engineered PNA molecules were used as an analogue of DNA for capturing DNA target for biosensor application. We compared the performance of PNA probes with DNA ones on the sensitivity and specificity to DNA target at the solution/electrode interface using the electrometer-based potentiometric biosensor. The lack of negative charges in the PNA backbone facilitates the hybridization with the DNA counterpart, reinforcing the interaction with the DNA target with shorter chain length. On the other hand, the lack of negative charges of the PNA probes reduces the electrostatic attraction of PNA-DNA duplex with the electrode surface, compromising the direct readout of captured DNA charges within the electrical double layer at the interface. Overall, there is no clear contrast between PNA and DNA probes for detecting DNA target of the length greater than 20-mer in the label-free potentiometry.

## Figures and Tables

**Table 1.** Sequences of the probes and targets used in the study.

**Entry**	**Sequences (5′to 3′)**
22-mer PNA & DNA probes	GCAAC CTTCC CTATT ACTCC AC
20-mer PNA & DNA probes	TACTG TGGTT ATTGC TGTCT
15-mer PNA & DNA probes	ATTCT GTCTT TCACG
22-mer target	CGTTG GAAGG GATAA TGAGG TG
20-mer target	ATGAC ACCAA TAACG ACAGA
15-mer target	TAAGA CAGAA AGTGC
22-mer non-target	AACGA TCTGG GTAGT CAAGA GC
20-mer non-target	AATGA GAATG GAACC GATGA
15-mer non-target	AATTT ACCTC CAGCT

**Table 2.** Lateral packing density of the PNA and DNA probes.

**Entry**	**Probe density, nm^−2^**
22-mer PNA/SAM	0.157 ± 0.037
20-mer PNA/SAM	0.225 ± 0.043
15-mer PNA/SAM	0.130 ± 0.022
20-mer DNA/SAM	0.076 ± 0.014
15-mer DNA/SAM	0.069 ± 0.012
